# Sex-Specific Skeletal Muscle Fatigability and Decreased Mitochondrial Oxidative Capacity in Adult Rats Exposed to Postnatal Hyperoxia

**DOI:** 10.3389/fphys.2018.00326

**Published:** 2018-03-29

**Authors:** Laura H. Tetri, Gary M. Diffee, Gregory P. Barton, Rudolf K. Braun, Hannah E. Yoder, Kristin Haraldsdottir, Marlowe W. Eldridge, Kara N. Goss

**Affiliations:** ^1^Department of Pediatrics, University of Wisconsin, Madison, WI, United States; ^2^Department of Kinesiology, University of Wisconsin, Madison, WI, United States; ^3^Department of Medicine, University of Wisconsin, Madison, WI, United States

**Keywords:** preterm birth, skeletal muscle, skeletal muscle metabolism, mitochondria, sex differences

## Abstract

Premature birth affects more than 10% of live births, and is characterized by relative hyperoxia exposure in an immature host. Long-term consequences of preterm birth include decreased aerobic capacity, decreased muscular strength and endurance, and increased prevalence of metabolic diseases such as type 2 diabetes mellitus. Postnatal hyperoxia exposure in rodents is a well-established model of chronic lung disease of prematurity, and also recapitulates the pulmonary vascular, cardiovascular, and renal phenotype of premature birth. The objective of this study was to evaluate whether postnatal hyperoxia exposure in rats could recapitulate the skeletal and metabolic phenotype of premature birth, and to characterize the subcellular metabolic changes associated with postnatal hyperoxia exposure, with a secondary aim to evaluate sex differences in this model. Compared to control rats, male rats exposed to 14 days of postnatal hyperoxia then aged to 1 year demonstrated higher skeletal muscle fatigability, lower muscle mitochondrial oxidative capacity, more mitochondrial damage, and higher glycolytic enzyme expression. These differences were not present in female rats with the same postnatal hyperoxia exposure. This study demonstrates detrimental mitochondrial and muscular outcomes in the adult male rat exposed to postnatal hyperoxia. Given that young adults born premature also demonstrate skeletal muscle dysfunction, future studies are merited to determine whether this dysfunction as well as reduced aerobic capacity is due to reduced mitochondrial oxidative capacity and metabolic dysfunction.

## Introduction

Currently, nearly 12% of infants are born preterm in the United States, defined as gestational age <37 weeks (Goldenberg et al., 2008; Frey and Klebanoff, 2016). Over the past few decades, advances in neonatal care have led to increasing numbers of children born at very low gestational ages surviving to adulthood (Younge et al., 2017). After early preterm birth, these infants generally require immediate resuscitation and treatment with life-sustaining oxygen therapy for prolonged periods. This exposure to a relatively hyperoxic environment at a time when the infant should still be *in utero* in a hypoxic environment has been associated with alterations of normal development and carries long-term consequences (Jobe and Kallapur, 2010; Raju et al., 2017a,b).

While early research investigating the consequences of premature birth traditionally focused on the pulmonary consequences in this population, other systemic long-term sequelae are increasingly well-documented. Children and young adults born preterm are shorter in stature, have higher systemic blood pressure, lower lean body mass, greater body fat mass, and lower systemic capillary density (Bonamy et al., 2007; Kajantie et al., 2010; Thomas et al., 2011; Parkinson et al., 2013; Crane et al., 2016). Multiple studies demonstrate a higher risk for metabolic syndrome, type 2 diabetes and insulin resistance, as well as fatty liver disease (Thomas et al., 2011; Sipola-Leppänen et al., 2015; Crane et al., 2016). In addition, teens and young adults born preterm have higher fasting insulin and augmented insulin release following a glucose load, consistent with increased insulin resistance (Hofman et al., 2004; Regan et al., 2006; Dalziel et al., 2007; Hovi et al., 2007; Pandolfi et al., 2008; Mathai et al., 2012; Li et al., 2014; Kajantie et al., 2015; Sipola-Leppänen et al., 2015; Morrison et al., 2016). Importantly, impaired skeletal muscle glucose disposal and skeletal mitochondrial dysfunction are key contributors to insulin resistance and metabolic disease (Kelley et al., 2002; Thyfault et al., 2006; Ritov et al., 2010; Jelenik and Roden, 2013; Petersen et al., 2015). Young adults born preterm also have evidence of a multifaceted decrease in exercise capacity, as shown by a lower aerobic capacity, lower maximal power output, and earlier anaerobic threshold (Vrijlandt et al., 2006; Duke et al., 2014; Farrell et al., 2015). Additionally, contractile dysfunction has been confirmed with direct measures of skeletal muscle capacity including reduced grip strength, earlier push up and sit up fatigue, and lower leg power testing in teens born preterm (Rogers et al., 2005).

Despite the mounting evidence of both skeletal muscle and glucose handling impairments after premature birth, the specific skeletal muscle metabolic alterations underlying these physiologic changes in preterm born adults have not been well-identified. In this study, we sought to identify alterations in essential regulators of glucose metabolism using postnatal hyperoxia exposure (HYP) in rats, a well-established model of bronchopulmonary dysplasia, or chronic lung disease of prematurity. Importantly, this animal model also recapitulates the pulmonary vascular, cardiovascular, and renal phenotype of premature birth (Yzydorczyk et al., 2008; Yee et al., 2009; Bavis et al., 2011; O'Reilly and Thébaud, 2014; Goss et al., 2017; Kumar et al., 2017). Although not a prematurity model *per se*, postnatal hyperoxia exposure in rats results in a relative hyperoxia exposure during a time of final organ development, at a developmental stage when rats have organ maturational stages similar to a 28–32 week infant(O'Reilly and Thébaud, 2014; Seely, 2017). We hypothesized that rats exposed to postnatal hyperoxia [Fraction inspired oxygen (F_I_O_2_) 0.85] for 14 days then aged to 1 year, or equivalent of mid-adulthood in humans, in standard conditions would have decreased skeletal muscle and mitochondrial function resulting in an increased compensatory glycolytic capacity in predominantly glycolytic muscles. A secondary aim was to evaluate sex differences after postnatal hyperoxia exposure.

## Materials and methods

### Animal model

Pregnant Sprague-Dawley dams (Harlan/Envigo, Indianapolis, Indiana) arrived a week before their due date and were allowed to deliver naturally. Within 12 h of birth, the pups from 4 litters were pooled and randomized to either hyperoxia (HYP, 85% oxygen) or normoxia (NORM, 21% oxygen) exposure in custom-made chambers (Coy Lab, Grass Lake, MI) for the first 14 days of life. Chambers were controlled for humidity, temperature, oxygen level, and carbon dioxide level. The dams were rotated between normoxia and hyperoxia daily to prevent maternal oxygen toxicity. Over the course of every 4 days, every litter had each dam for 24 h, to ensure equal maternal treatment of all litters.

After 14 days, the pups were removed from the chambers and aged to 1 year in room air. Pups were weaned per standard husbandry at day 24. At 1 year, the animals used for molecular analysis were sacrificed by urethane (*n* = 12 per group, 6 males), and the gastrocnemii were dissected. The right lateral gastrocnemius was flash frozen in liquid nitrogen and the right medial gastrocnemius was fixed in formalin. Animals used for mitochondrial respiration measurements, electron microscopy, and muscle fatigue measurements were euthanized by isofluorane and cervical dislocation (NORM *n* = 11, 5 male; HYP *n* = 10, 5 male). In these animals, the plantaris was dissected for mitochondrial respiration measurements, the central section of the medial gastrocnemius was fixed for electron microscopy, and the flexor digitorum brevis was dissected and used for measurements of muscle fatigue. The gastrocnemius was chosen as a mixed muscle with fast twitch predominance, and for its large size which allowed for more analyses with the same muscle. The plantaris was chosen for mitochondrial respiration due to its more homogenous fiber type distribution, to reduce differences due to sampling. The flexor digitorum brevis was chosen as it is a smaller predominantly fast twitch muscle and thus the force would not exceed the maximal force possible to measure with the force transducer.

All animal handling was approved by the UW Institutional Animal Care and Use Committee and conducted in accordance with the guidelines in the National Institutes for Health “Guidelines for the Care and Use of Laboratory Animals, 8th Edition.”

### Histologic analysis

The medial lobe of the gastrocnemius was fixed in paraformaldehyde and embedded in paraffin. The gastrocnemius was sectioned across the belly of the muscle and stained with hematoxylin and eosin. Images were then taken using a 20X magnification objective lens across the middle of each medial gastrocnemius cross-sectional slices. Muscle fiber cross sectional area was quantified using ImageJ.

### Western blotting

The flash frozen gastrocnemius was powderized in liquid nitrogen with a mortar and pestle to ensure homogenous sampling for molecular experiments. Protein was isolated in 10 μl RIPA buffer (Sigma-Aldrich, Saint Louis, Missouri) per mg of tissue with 1:1,000 phosphatase inhibitor cocktail (Sigma-Aldrich, Saint Louis, Missouri) and 1:100 protease inhibitor cocktail (Sigma-Aldrich, Saint Louis, Missouri). Protein concentration was measured by Bradford assay (Sigma-Aldrich, Saint Louis, Missouri). Thirty micrograms of protein was denatured at 95°C for 5 min and loaded into 4–12% Bis-Tris Novex NuPage gels (Thermo-Fisher Scientific, Waltham, Massachusetts). After electrophoresis, protein was transferred to nitrocellulose membranes and sequentially incubated with 5% bovine serum albumin, primary antibodies, and secondary antibodies. The primary antibodies used were GLUT4 (Abcam, Cambridge, UK), HKI (Abcam, Cambridge, UK), HKII (Cell Signaling Technology, Danvers, MA), PFK1 (Santa Cruz Biotechnology, Dallas, TX), PDH (Abcam, Cambridge, UK), LDHA (Santa Cruz Biotechnology, Dallas, TX), CS (Abcam, Cambridge, UK), complexes I-V cocktail (containing CI subunit NDUFB8, CII-30kDa, CIII core protein 2, CIV subunit I, and CV alpha subunit) (Abcam, Cambridge, UK), PGC1α (Calbiochem, Millipore, Billerica, MA), TFAM (Abcam, Cambridge, UK), Mfn1 (Abcam, Cambridge, UK), Mfn2 (Cell Signaling Technology, Danvers, MA), Drp1 (Abcam, Cambridge, UK), Fis1 (Sant Cruz Biotechnology, Dallas, TX), and OPA1 (Abcam, Cambridge, UK). Vinculin was used a loading control (Calbiochem, Millipore, Billerica, MA). Blots were imaged using near infrared imaging (LiCor, Lincoln, NE) which is linear and thus allows more accurate quantification than standard methods. Images were quantified with ImageStudio (Licor, Lincoln, NE).

### Enzyme activity assays

To assess citrate synthase activity, tissue was homogenized in CelLytic Mt Cell Lysis Reagent (Sigma-Aldrich, Saint Louis, Missouri) with protease inhibitors (Sigma-Aldrich, Saint Louis, Missouri) and the activity level was measured using a colorimetric Citrate Synthase Activity Assay (Sigma-Aldrich, Saint Louis, Missouri). Specific activity was calculated as relative to protein mass, as measured by Bradford Assay.

Complex I and complex II activity levels were measured using immunocapture colorimetric assays (Abcam, Cambridge, Massachusetts); samples were prepared according to directions after having been flash frozen upon dissection. Specific activity was calculated as relative to protein mass, as measured by Bradford Assay.

### Mitochondrial respiration measurements

The right plantaris was removed from the right hindlimb and immediately placed in ice cold BIOPS solution (10 mM Ca-EGTA, 0.1 μM free calcium, 20 mM imidazole, 20 mM taurine, 50 mM K-MES, 0.5 mM DTT, 6.56 mM MgCl_2_, 5.77 mM ATP, 15 mM phosphocreatine, pH 7.1) (Pesta and Gnaiger, 2011). Bundles of fibers weighing approximately 10 mg were dissected out and the individual fibers were teased apart into a mesh-like bundle. The fibers were permeabilized in BIOPS with 0.005% saponin (w/v) for 30 min on ice. Permeabilized fibers were then washed in MiR05 respiration media (0.5 mM EGTA, 20 mM taurine, 3 mM MgCl_2_, 110 mM sucrose, 60 mM K-lactobionate, 10 mM KH_2_PO_4_, 20 mM K-HEPES, 1 mg/ml BSA, pH 7.1) for 10 min on ice.

Oxygen concentration was measured, and oxygen flux was calculated using an Oxygraph-2k (Oroboros Instruments, Innsbruck, Austria) and its software, DatLab7.0. Permeabilized muscle fiber respiration was measured in MiR05 in the Oxygraph-2k at 37°C with constant stirring. To assess proton leak, glutamate (10 mM) and malate (5 mM) were added to create a high substrate, low ADP state (state 4). Next, ADP (5 mM) was added to assess complex I activity (state 3). Complex I was inhibited with rotenone (0.5 μM) and complex II was stimulated with succinate (10 mM). Complex III was inhibited with antimycin A (5 μM). Complex IV was assessed with an artificial substrate, N,N,N′,N′-tetramethyl-p-phenylenediamine dihydrochloride (TMPD) (2 mM), which acts as an electron donor to cytochrome c, and ascorbate (8 mM) which prevents TMPD auto-oxidation. Carbonyl cyanide m-chlorophenyl hydrazone (CCCP) (0.5 μM) was used to achieve a non-coupled state for measuring maximal electron transport system (ETS) capacity. Oxygen flux was normalized to wet tissue weight (Kuznetsov et al., 2008).

### Fatigue testing

The entire right flexor digitorum brevis (FDB) was excised and placed in oxygenated room temperature Tyrode's solution (120 mM NaCl, 5 mM KCl, 2 mM CaCl_2_, 0.5 mM MgCl_2_, 0.4 mM NaH_2_PO_4_, 24 mM NaHCO_3_, 0.1 EDTA, 10 mM glucose). The FDB was then attached at the distal and proximal tendons to a force transducer and length controller (Aurora Scientific), stretched to optimal length, and stimulated with platinum electrodes starting at 10V with increasing potential until maximal tetanic force was achieved. The FDB was stimulated at 100 Hz for 500 ms every 5 s. The force from eight tetanic stimulations were averaged to assess maximal tetanic force production. The FDB underwent continuous stimulation for 10 min, and then the maximal tetanic force was again measured. Force after 10 min of continuous stimulation was calculated as a percent of maximum force, and this was termed the “fatigue force.” The FDB was allowed to recover for 20 min with no stimulation and force was once again measured to ensure that the muscle remained viable.

### Electron microscopy

Central sections of the right lateral gastrocnemius were dissected into strips with a cross-sectional area of approximately 1 mm^2^ and immersion fixed in 0.1M sodium cacodylate buffer with 2.5% glutaraldehyde, and 2% paraformaldehyde, at least overnight in 4°C. After primary fixation, samples were loaded into an mPrep System ASP-1000 Specimen Processor (Microscopy Innovations, LLC, Marshfield, WI) which automated post-fixation in 1% osmium tetroxide in the same buffer. Samples were then dehydrated in a graded ethanol series followed by rinses in 100% acetone and finally, the samples were infiltrated and embedded in Embed 812 epoxy resin. All automated steps were carried out in mPrep tissue capsules.

Samples were sectioned for transmission electron microscopy using a Leica EM UC6 Ultramicrotome and contrasted with 8% uranyl acetate in 50% ethanol and Reynolds lead citrate. Ultrathin sections were observed with a Philips CM120 electron microscope and images were captured with an AMT BioSprint side mounted digital camera using AMT Capture Engine software. Each sample had multiple cells imaged at 4400x, 15,000x, and 31,000x magnification for assessment of mitochondria content and quality.

### Statistics

Comparisons were made by two-way ANOVA with Tukey's honest statistical difference for multiple comparisons. Tests were run with GraphPad Prism (GraphPad, La Jolla, CA), and a p value < 0.05 was considered significant. *p*-values less than or equal to 0.09 are reported. Data points with a modified z score > 3.5 were excluded.

## Results

### Body composition and muscle composition

**Figure 1:**
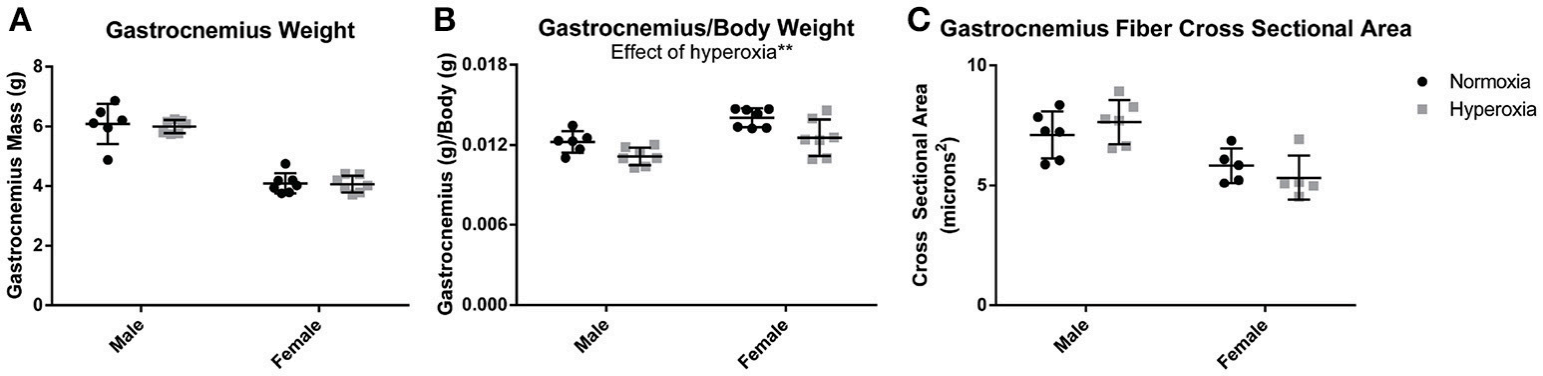
Muscle mass and fiber size. There was no difference in gastrocnemius mass between HYP and NORM rats of the same sex **(A)**. There was a lower gastrocnemius mass to body mass ratio, suggesting a higher body fat mass **(B)**. There was no difference in fiber cross sectional area, indicating a lack of hypertrophy or atrophy of the gastrocnemius **(C)**. ^**^p < 0.01.

Gastrocnemius weight (Figure 1A) and soleus weight (data not shown) were similar between aged HYP and control (NORM) rats of each sex. Despite 8% higher body mass in HYP males and 12% higher body mass in HYP females (animal weights previously published) (Goss et al., 2017), HYP rats had significantly reduced gastrocnemius to body weight ratio (9% lower in HYP males, and 11% lower in HYP females) (Figure 1B) and soleus to body weight ratio (data not shown). This lower muscle mass to total body mass ratio suggests a larger fat mass in HYP rats, despite similar muscle mass overall. Light microscopy examination of the gastrocnemius was similar between HYP rats and NORM rats (H&E images and Masson's trichrome images not shown). There were no differences in fiber cross-sectional areas across any of the groups (Figure 1C), suggesting absence of atrophy.

### Muscle function and mitochondrial respiration

**Figure 2:**
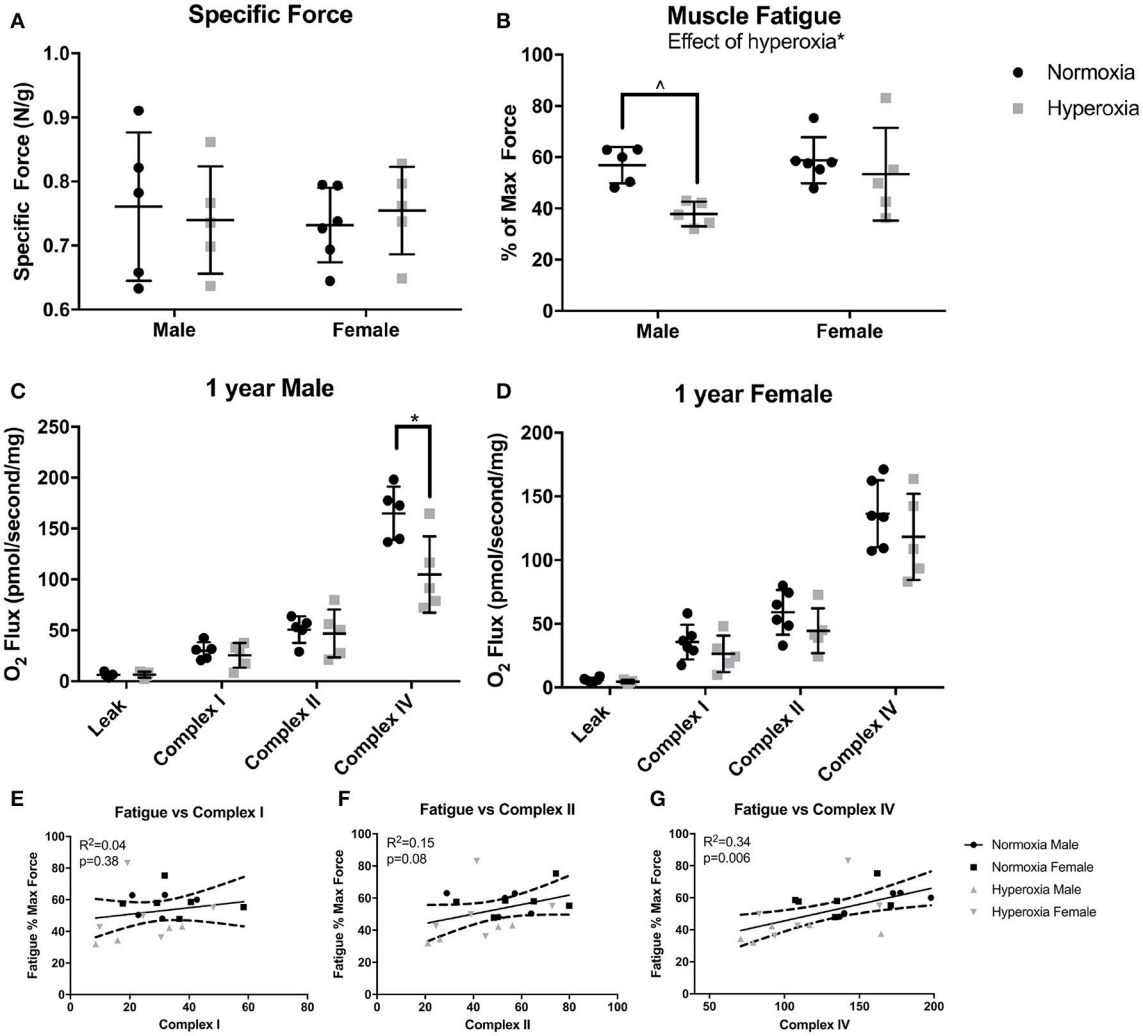
Muscle and mitochondrial function. There were no differences in specific force between NORM and HYP rats **(A)**. However, there was lower fatigued force in the HYP rats, with the difference driven by the large reduction in HYP male rats **(B)**. There was lower complex IV supported respiration in HYP male rats compared to NORM male rats, with no differences in complex I supported respiration and complex II supported respiration **(C)**. There were no differences in mitochondrial respiration between HYP and NORM female rats **(D)**. Muscle fatigue correlated best with complex IV supported respiration **(E–G)**. ^∧^0.09 > *p* > 0.05, ^*^p < 0.05.

To assess muscle function, maximal force production and muscle fatigue were measured in the flexor digitorum brevis muscle, a predominantly fast twitch muscle. Fatigue force is the maximal tetanic force after 10 min of continuous stimulation reported as a percent of the initial maximal force produced. There were no differences in initial maximal specific force (N/mg) between HYP and NORM rats (Figure 2A). An effect of hyperoxia treatment on muscle fatigue (*p* = 0.02) was present, however, the increased fatigue was predominantly driven by male HYP rats. The flexor digitorum brevis fatigued to a greater degree in HYP males than in NORM males (37.8 ± 5.5 vs. 56.9 ± 7.2 percent of initial maximum force, *p* = 0.058) demonstrating muscle dysfunction in this population. There was little difference in fatigability in the HYP females compared to NORM females (53.4 ± 18 vs. 58.8 ± 9.0 percent of initial maximum force, *p* = 0.86) (Figure 2B).

Mitochondrial respiration in permeabilized fibers from the plantaris muscle showed no differences in the respiratory control ratio (RCR) (ratio of complex I, high substrate, ADP stimulated respiration to high substrate, ADP absent respiration), in leak state, nor complex I or complex II supported respiration between HYP males and NORM males (Figure 2C). However, there was a significant effect of hyperoxia on complex IV supported respiration (*p* = 0.01). The HYP males had 37% lower complex IV supported respiration compared to NORM males (104.6 ± 37.8 vs. 165.0 ± 26.2 pmol/s/mg, respectively, *p* = 0.03) (Figure 2C). Furthermore, respiration was not significantly increased by addition of CCCP, indicating that the measurements of complex IV reflected maximal complex IV function and were not limited by ATP synthase capacity (data not shown). In the HYP females, there were no differences in RCR, leak state respiration, or complex I or II respiration compared to NORM females (Figure 2D). Of note, the mean RCR values for each group ranged from 4.2 to 7.6, as is similar to many studies in glycolytic predominant muscle (Min et al., 2011; Joseph et al., 2013; Morrow et al., 2017). While some methods state that RCRs should range from 7 to 9 in glycolytic predominant muscle, it has also been well-documented that RCRs decrease with age (Chabi et al., 2008; Kuznetsov et al., 2008; Johnson et al., 2015).

Correlating fatigability results with specific mitochondrial complex activity can help identify specific defects in mitochondrial respiratory chain that may be one of the potential factors contributing to increased fatigue. Across all the animals, complex I capacity did not correlate with muscle fatigue (R^2^ = 0.04, *p* = 0.48). Complex II capacity also did not correlate with fatigue (R^2^ = 0.15, *p* = 0.08). Interestingly, fatigue correlated most strongly with complex IV capacity (R^2^ = 0.34, *p* = 0.006) (Figures 2E–G).

**Figure 3:**
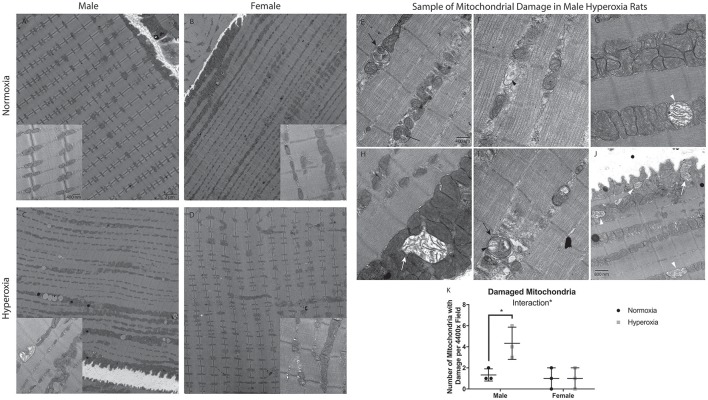
Electron micrographs of gastrocnemius, at either 4400x or 31,000x magnification, with the exception of J at 25,000x magnification. NORM male and female rats had evenly spaced mitochondria, with healthy structure and organization **(A,B)**. HYP male rats had less organized structure and location of the mitochondria. Further, there were many mitochondria with swelling, vacuolization, and membrane damage **(C)**. HYP female rats had no apparent differences to their mitochondria **(D)**. Representative examples of HYP male mitochondrial damage are shown. The skinny black arrow points to an example of vacuolization. Black arrowhead indicates examples of cristae damage. Black arrow indicates sample myelin bodies. The white arrowhead indicates mitochondrial swelling. The filled in white arrow indicates mitochondria with membrane damage **(E–J)**. HYP male rats have significantly more damaged mitochondria than NORM males, with no difference in female groups, resulting in a significant interaction of sex and treatment **(K)**. ^*^p < 0.05.

Given the significant mitochondrial dysfunction, electron microscopy was used to evaluate for mitochondrial structural abnormalities. The mitochondria had an overall disorganized appearance in both structure and localization when compared to NORM males (Figures 3A–D). HYP males had many mitochondria with swelling, vacuolization, as well as cristae and membrane damage (Figures 3E–J). In contrast, there were no major differences observed in mitochondrial structure in the HYP females compared to NORM females. There was a significant interaction between sex and treatment with HYP males having a greater number of damaged mitochondria per field than NORM males, with no differences between the female groups (Figure 3K).

### Oxidative phosphorylation enzyme expression and activity

**Figure 4:**
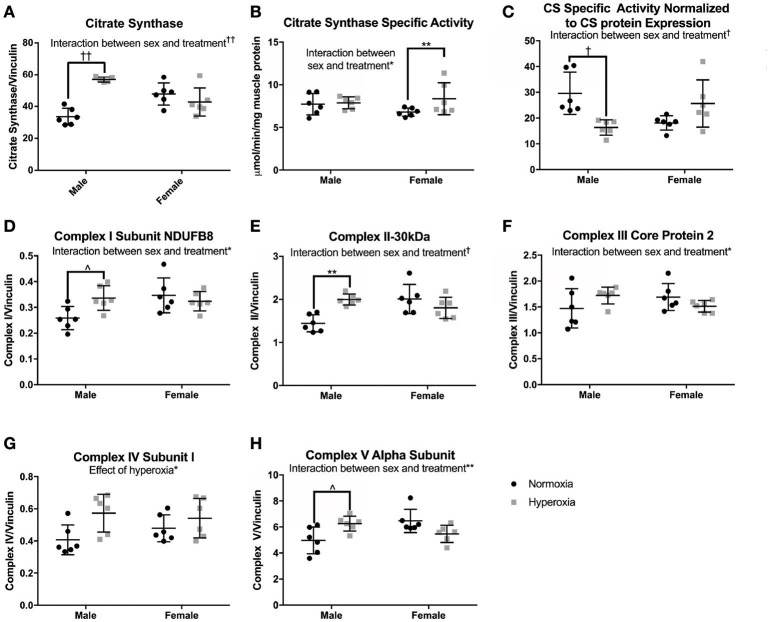
Markers of mitochondrial mass. HYP male rats had increased protein expression of markers of mitochondrial mass **(A,D–H)**, yet no difference in CS specific activity **(B)**, and decreased CS specific activity normalized to CS protein expression **(C)**, whereas HYP females did not. ^∧^0.09 > *p* > 0.05, ^*^p < 0.05, ^**^p < 0.01, ^†^*p* < 0.001, ^††^*p* < 0.0001.

To assess skeletal muscle mitochondrial mass and TCA cycle function, citrate synthase (CS) protein expression, and activity were measured. CS protein expression showed a significant interaction between sex and treatment, with a higher level in HYP males than NORM males (69% higher) but no difference between the female treatment groups (Figures 4A–C). CS specific activity level also had a significant interaction effect with no difference between male treatment groups and a significantly higher CS activity level in the HYP females compared to the NORM females (23% higher). When analyzed for specific activity compared to CS expression by western blot to assess for the functional activity per expression, there was a significant interaction with HYP males having a significantly lower CS specific activity relative to CS expression compared to NORM males (45% lower) and no difference between the female treatment groups.

As additional markers of mitochondrial mass, Complexes I (subunit NDUFB8), II (−30 kDa), III (core protein 2), and V (alpha subunit) were measured and all had significant interactions between sex and treatment groups for protein expression (Figures 4D–H; Supplemental Figure 1). There were no significant differences in protein expression of these complexes between the female groups, yet HYP males showed higher expression of complexes I, II, III, and V compared to NORM males, suggesting greater total mitochondrial mass. Complex IV (subunit I) protein expression was significantly higher in all HYP rats compared to controls (29% higher in HYP males, and 13% higher in HYP females).

Finally, to evaluate mitochondrial respiration in a second fast-twitch muscle, we assessed complex I and II activity by immunocapture in the gastrocnemius. There were no differences in complex I specific activity level or specific activity relative to complex I expression with regard to sex or postnatal exposure. There was a trend toward lower complex II specific activity relative to complex II expression in the HYP males compared to NORM males (*p* = 0.07), but no difference in complex II specific activity (data not shown). This immunocapture activity data in the gastrocnemius resembles the mitochondrial respiration data in the plantaris.

### Mitochondrial biogenesis and dynamics

**Figure 5:**
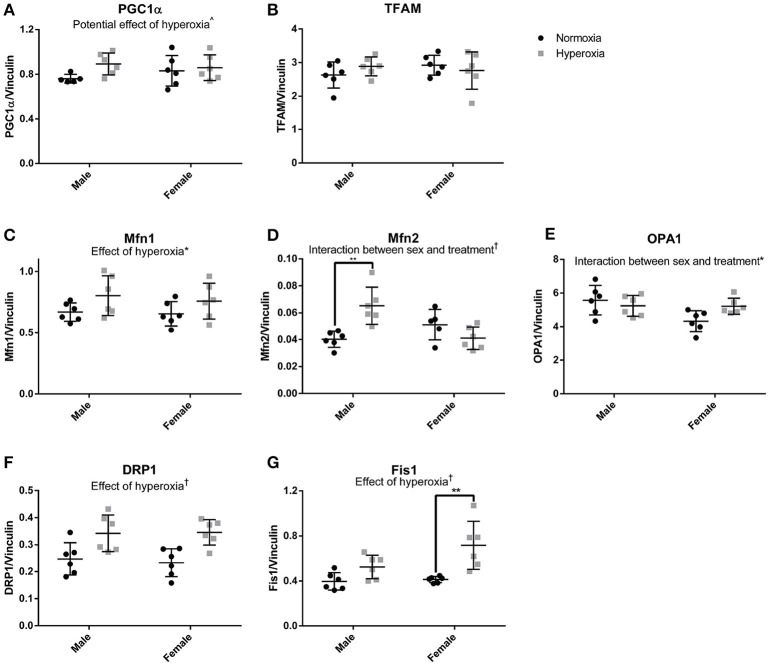
Markers of mitochondrial dynamics. There were no statistical differences in markers of mitochondrial biogenesis **(A,B)**. HYP male rats had increased expression of proteins involved mitochondrial fusion, but not HYP female rats **(C–E)**. HYP rats had increased markers of mitochondrial fission **(F,G)**. ^∧^0.09 > *p* > 0.05, ^*^p < 0.05, ^**^p < 0.01, ^†^*p* < 0.001.

To assess the role of mitochondrial biogenesis and turnover in the decreased skeletal muscle function and mitochondrial respiration in HYP males, we examined the transcriptional control of mitochondrial turnover. There were no differences in the molecular markers of mitogenesis as assessed by peroxisome proliferator-activated receptor gamma coactivator 1 alpha (PGC1α) and mitochondrial transcription factor A (TFAM) expression (Figures 5A,B; Supplemental Figure 1).

However, there were significant differences in markers of mitochondrial fusion. Mitofusin 1 (Mfn1), a protein involved in outer membrane fusion, showed significantly higher expression in HYP rats compared to controls (20% higher in HYP males, and 16% higher in HYP females). Mitofusin 2 (Mfn2), another protein required for outer membrane fusion, had a significant interaction between sex and treatment, with higher expression in HYP males compared to NORM males (62% higher) but no differences between female treatment groups. Dynamin-like 120 kDA protein (OPA1), a regulator of inner membrane mitochondrial fusion, also had a significant interaction between sex and treatment, with no differences in the males between treatment groups, and lower expression in the HYP females compared to NORM females (Figures 5C–E; Supplemental Figure 1).

Dynamin-related protein 1 (Drp1) and mitochondrial fission 1 protein (Fis1), both part of the machinery involved in mitochondrial fission, had significantly higher expression in HYP rats compared to NORM rats. Drp1 was 38% higher in HYP males and 48% higher in HYP females whereas Fis1 was 32% higher in HYP males, and 74% higher in HYP females (Figures 5F,G; Supplemental Figure 1). Overall, these results suggest increased dynamic turnover of the mitochondria in the absence of active biogenesis.

### Changes in glycolytic enzyme expression

**Figure 6:**
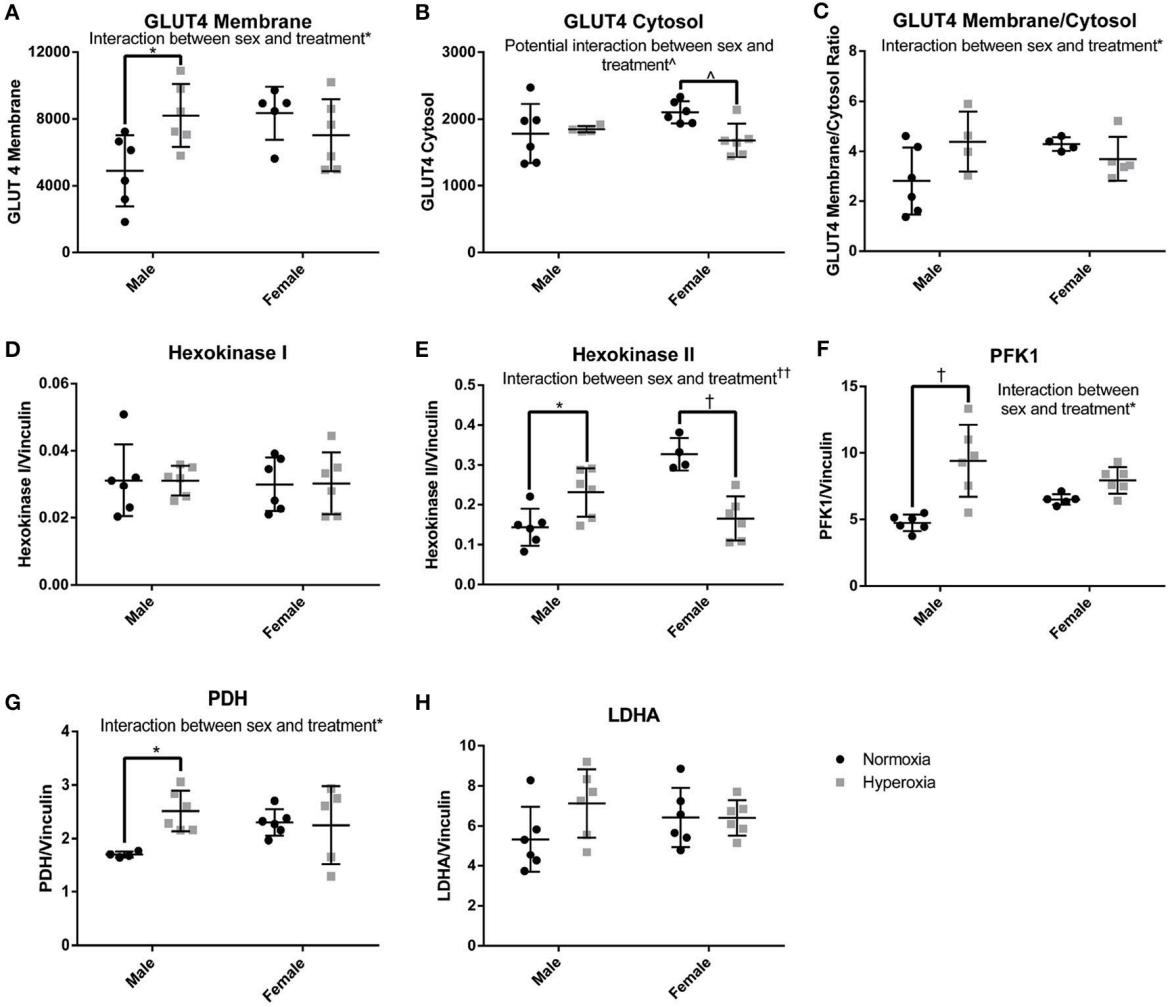
Glucose transport and glycolysis. HYP male rats had increased levels of proteins involved in glucose transport into the myocyte **(A–C)**, no difference in expression of constitutively present isoform of hexokinase **(D)** and increased expression of key regulated glycolytic enzymes **(E–G)**. There was no difference in LDHA expression **(H)**. ^∧^0.09 > *p* > 0.05, ^*^p < 0.05, ^†^*p* < 0.001, ^††^*p* < 0.0001.

In order to determine if there was a compensatory response to the mitochondrial defects identified in HYP rats with alterations in anaerobic glucose metabolism, the protein expression of key regulated steps in glucose transport and glycolysis were measured. In the gastrocnemius of HYP rats, there was a significant interaction between sex and treatment group in protein expression of glucose transporter type 4 (GLUT4), a striated muscle and adipose insulin-dependent glucose transporter. HYP males had significantly higher membrane expression of GLUT4 than NORM males (68% higher) and no difference in the cytosolic content of GLUT4 protein. The GLUT4 membrane to cytosol ratio thus had a significant interaction with a higher ratio in the HYP males compared to NORM males, while there were no significant differences between HYP and NORM females (Figures 6A–C; Supplemental Figure 1).

By phosphorylating glucose transported into cells, the hexokinases play a key role in glucose disposition by committing glucose to intracellular metabolism or storage rather than efflux out of the cell. The expression of hexokinase I, the constitutively present isoform, was similar between groups. However, there was a significant interaction between sex and treatment for the expression of hexokinase II, the regulated isoform. Hexokinase II was significantly higher in HYP male rats compared to NORM male rats (61% higher), yet significantly lower in HYP female rats compared to NORM female rats (50% lower) (Figures 6D,E; Supplemental Figure 1).

Phosphofructokinase 1 (PFK1) also plays a key role in glucose metabolism by committing glucose to the glycolytic pathway. PFK1 protein expression showed an interaction between sex and treatment. PFK1 was significantly higher in HYP male rats compared to NORM male (98% higher), yet no statistically significant differences were found in PFK1 between the female groups. Pyruvate dehydrogenase, the key enzyme committing pyruvate to a mitochondrial fate, showed a similar expression profile to PFK1 (HYP males have 48% higher expression than NORM males) (Figures 6F–H; Supplemental Figure 1). There were no differences in lactate dehydrogenase expression.

## Discussion

Adolescents and adults with a history of preterm birth have decreased exercise capacity and an increased prevalence of metabolic diseases such as type 2 diabetes mellitus, hepatic fat deposition, and obesity (Rogers et al., 2005; Vrijlandt et al., 2006; Thomas et al., 2011; Duke et al., 2014; Farrell et al., 2015; Kato et al., 2015; Crane et al., 2016; Morrison et al., 2016). While these systemic functional differences are known, there is limited molecular or cellular data to explain the effects of preterm birth and its associated environmental exposures such as postnatal oxygen therapy on this increased risk of metabolic disease. Skeletal muscle plays an important role in glucose disposition and defects in skeletal muscle function and insulin responsiveness are associated with an increased risk of metabolic disease (Samuel and Shulman, 2016). Both contractile and metabolic muscle function are dependent upon normal mitochondrial activity. Given that postnatal hyperoxia exposure in rats recapitulates many of the phenotypic aspects of premature birth, we hypothesized that rats exposed to postnatal hyperoxia would have a reduction of muscle function due to impaired mitochondrial function with glycolytic compensation.

In this study, we used postnatal hyperoxia exposure to induce a phenotype mimicking premature transition to a relative hyperoxia exposure that premature infants encounter. Rats are born relatively premature compared to humans at term, with lungs and other organs roughly equivalent to those of a 28 week human fetus (O'Reilly and Thébaud, 2014; Seely, 2017). However, rats develop normally in room air. Thus, to mimic the relative hyperoxia that preterm infants experience, the HYP model exposes rats to hyperoxia continuously for 14 days after birth. Postnatal hyperoxia is a well-accepted rodent model of lung disease associated with preterm birth (O'Reilly and Thébaud, 2014) and multiple other studies have shown the HYP model to reproduce phenotypic features of adults who were born premature and exposed to postnatal hyperoxia, including pulmonary vascular disease, impaired ventilatory control, cardiovascular disease, decreased systemic vascular density, renal insufficiency and fibrosis, and stunted pulmonary development (Yzydorczyk et al., 2008; Bavis et al., 2011; Yee et al., 2011; O'Reilly and Thébaud, 2014; Goss et al., 2017; Kumar et al., 2017).

In the experiments described here, the HYP rats had increased body weight without an increase in muscle mass at 1 year, likely indicating an increased fat mass. A few studies of human adults born preterm show an increased fat mass compared to adults born term (Thomas et al., 2011; Sipola-Leppänen et al., 2015; Crane et al., 2016; Morrison et al., 2016), while other studies show no difference in fat mass (Hovi et al., 2007; Rotteveel et al., 2008; Smith et al., 2011; Kerkhof et al., 2012; Kajantie et al., 2015). One difference between our rat model and studies of humans born preterm is that we did not find a difference in muscle mass, likely indicating no differences in lean body mass, whereas most studies have shown lower lean body mass in adults born premature (Hovi et al., 2007; Kajantie et al., 2015; Morrison et al., 2016). However, other studies demonstrate no differences in lean body mass (Smith et al., 2011; Sipola-Leppänen et al., 2015). It is possible that these differences observed in body composition in human studies are related to differences in care and nutrition in the neonatal period, or lifelong differences in physical activity and diet.

In the male HYP rats, we found decreased skeletal muscle function, as measured by fatigability. Our finding of higher muscle fatigue in adult male HYP rats aligns well with the decreased exercise capacity observed in preterm born adolescents and adults described in the literature (Rogers et al., 2005; Vrijlandt et al., 2006; Duke et al., 2014; Farrell et al., 2015). We found no difference in specific force accompanied by increased muscle fatigue in male HYP rats, a finding that we interpret to mean the alterations in contractile function are not at the myofilament level but are due to disruptions in metabolism and energy production. Our finding of increased fatigability in male HYP rats is similar to findings in humans. While multiple studies have shown decreased aerobic exercise capacity and maximal power output in adult humans born preterm,(Vrijlandt et al., 2006; Duke et al., 2014; Farrell et al., 2015), aerobic capacity is not only influenced by skeletal muscle metabolism but also by cardiorespiratory fitness and function. More directly similar to our findings in HYP rats, Rogers et al. found greater muscle fatigue in adolescents born premature, as assessed by decreased push up and sit up (Rogers et al., 2005). Unfortunately, the aforementioned studies did not analyze their data for sex and birth status interactions. The finding that male HYP rats exhibit increased skeletal muscle fatigability but the female HYP rats do not is supported by an unpublished *post-hoc* analysis of the data reported in Farrell et al. (2015). This analysis shows reduced exercise capacity in preterm born adults was driven solely by preterm-born males. Specifically, maximal power output by adult males born preterm was significantly lower compared to control males (222 ± 30 vs. 292 ± 38 W respectively, *p* = 0.031), but not in females (176 ± 28 vs. 170 ± 37 W respectively, *p* = 0.87). The *post-hoc* analysis of the albeit small Farrell et al. study demonstrates that the differences in exercise capacity between term and preterm born adults are driven by a markedly reduced exercise capacity of males, whereas females appear to be unaffected. This would be consistent with prior research demonstrating greater clinical impairments among preterm males than females in early childhood, and suggests that this difference may translate into clinically meaningful differences in cardiorespiratory function in adulthood (Thomas et al., 2011; Peacock et al., 2012). Because impaired cardiorespiratory fitness is an independent predictor of cardiovascular mortality and morbidity, preterm born males may be at greater risk than females (Lee et al., 2010). These findings in the literature and this study show that there is great need to further study sex differences in metabolic and cardiorespiratory outcomes in adult survivors of preterm birth.

While there are no studies looking at mitochondrial respiration capacity in muscle biopsies of human adults with a history of preterm birth, there is evidence in the literature demonstrating reduced mitochondrial respiratory capacity in skeletal muscle of infants born preterm (Wenchich et al., 2002; Honzik et al., 2008). Our data suggests that this impaired mitochondrial function could persist into adulthood. In the male HYP rats at 1 year, we found lower maximal ETS capacity. We observed a 37% decreased complex IV stimulated respiration. Due to the published quantitative relationship between Complex IV activity and Complex I stimulated respiration, this level of complex IV dysfunction would be expected to result in an approximately 10% decrease in overall mitochondrial respiratory capacity *in vivo* (Letellier et al., 1994; Rossignol et al., 1999). Mitochondrial respiratory capacity has been observed to correlated with walking speed, muscular VO_2_ and whole body VO_2_ in untrained individuals (Coen et al., 2013; Gifford et al., 2016). It is therefore likely that the decrease in mitochondrial respiration may contribute to the observed increased fatiguability in our model. The relationship between mitochondrial respiration and skeletal muscle function in this model, as well as in humans with a history of premature birth, is an area that requires further study in the future. In addition to impaired mitochondrial ETS function, there were also mitochondrial morphological changes with vacuolization, swelling, and membrane and cristae damage in the male HYP rat muscles. This mitochondrial damage has been reported to be associated with loss of membrane potential and activation of mitochondrial permeability transition, often occurring with hypoxic insult and the initiation of apopotosis (Di Lisa and Bernardi, 1998; Sesso et al., 2012). The presence of such pathologic changes may be indicative of a biochemically sensitive or stressed organelle with little ability to compensate for additional stress occurring during the tissue procurement. While there is no data examining mitochondrial function and damage in adults with a history of preterm birth, there is significant data showing lower mitochondrial respiration and similar mitochondrial damage in humans with insulin resistance as well as a large number of studies showing insulin resistance in the preterm population (Kelley et al., 2002; Ritov et al., 2010; Mathai et al., 2012; Morrison et al., 2016). Interestingly, others have demonstrated a protective role of estrogen in maintaining skeletal muscle mitochondrial quantity and function that is lost with ovariectomy of female rats,(Cavalcanti-de-Albuquerque et al., 2014), suggesting that estrogen could have a protective effect in our female HYP rats that was absent in the male HYP rats.

The molecular characterization of skeletal muscle mitochondrial markers and glucose metabolism in the HYP rats further supports the muscle dysfunction that we observed. Citrate synthase protein expression is often used as a marker of mitochondrial volume and in healthy skeletal muscle, complex II and V protein expression levels also correlate well with mitochondrial content. Complex III and IV have a weaker, but still significant correlation with mitochondrial content (Larsen et al., 2012). CS activity and ETS activity have also been correlated with mitochondrial content in healthy tissue (Larsen et al., 2012). In our experiments, the HYP male rats showed increased expression of CS, in addition to increased expression of ETS proteins indicative of increased mitochondrial mass. However, the HYP rats demonstrated an unexplained lack of correlation between CS expression and CS function, a finding that raises the possibility that CS function may not be an accurate measure of mitochondrial volume in disease states. These observed changes are likely indicative of a state of mitochondrial dysfunction partially compensated by increased mitochondrial mass. Although mitochondrial biogenesis markers were not increased, there were significantly higher levels of proteins necessary for mitochondrial fission and fusion. Intriguingly, decreased mitochondrial dynamics are described in most studies of insulin resistance (Montgomery and Turner, 2015). However, increased markers of mitochondrial dynamics have been described as essential for initiating apoptosis and for controlling mitochondrial damage (Suen et al., 2008).

Compensatory increases in glycolytic capacity have been observed in skeletal muscle biopsies of patients with type 2 diabetes, which, as described above, is known to have increased prevalence in the preterm population (Simoneau and Kelley, 1997; Oberbach et al., 2006). We hypothesized that we might find evidence of increased glycolytic capacity in our HYP rats. In male HYP rats, we found an increase of all regulated steps of glycolysis, including the GLUT4 membrane to cytosol ratio, HK II, PFK1, and PDH protein levels. None of these alterations were observed in the female HYP rats. Thus, the mitochondrial dysfunction found in HYP male rats may be partially compensated by an increase in glycolytic and glucose transport protein expression.

A possible limitation of this study is the use of a rodent model of postnatal hyperoxia. The choice of a rodent postnatal hyperoxia exposure was based on prior studies that have characterized this model demonstrating similar pathological and physiological consequences as in human prematurity and the associated postnatal hyperoxia exposure that these neonates undergo (Yzydorczyk et al., 2008; O'Reilly and Thébaud, 2014; Goss et al., 2017). *In utero*, fetuses exist in a hypoxic environment and preterm birth exposes these infants to a normoxic environment, which is relatively hyperoxic compared to the *in utero* environment. In addition to the early exposure to room air, many of these infants also are given supplemental oxygen to allow them to maintain a blood oxygen content that is adequate to prevent end organ damage (Tin and Gupta, 2007). As the rat pups are born with relatively immature organs, the HYP model takes an immature host and exposes it to a relative hyperoxia, thus mimicking preterm birth with oxygen therapy (O'Reilly and Thébaud, 2014; Seely, 2017). While this model does not perfectly mimic premature birth, it does allow for a hyperoxic exposure in an immature organism. The data generated by this model provides future directions for study in human cohorts.

Another possible limitation of this study was the use of multiple different muscles. Muscles with mixed fiber types that are known to be primarily fast-twitch with higher glycolytic capacity were used based on a study showing the presence of high levels of lipid in the tibialis anterior, a predominantly fast twitch muscle, and not the soleus, a predominantly slow twitch muscle in preterm born adults (Thomas et al., 2011). Initially, the gastrocnemius was chosen for its mixed oxidative and glycolytic profile; however, due to the heterogeneous nature of the gastrocnemius fiber type distribution, it may not be an ideal choice for studies of mitochondrial respiration where sampling could provide samples with variable respiration capacity. The plantaris was chosen as it is a more homogenously mixed, albeit more glycolytic muscle. Unfortunately, the size of the gastrocnemius and plantaris made their use implausible for the fatigue measurements, thus a smaller primarily glycolytic muscle, the flexor digitorum brevis, was chosen. While this is a limitation of this study, the data was consistent across the different glycolytic predominant lower hindlimb muscles.

Our study shows that rats exposed to postnatal hyperoxia develop systemic effects extending to the skeletal muscle. Not only do our results replicate results observed in the human population born premature and thus exposed to postnatal hyperoxia such as muscle fatigability, but we also show that in this rodent model, muscle dysfunction correlates with mitochondrial dysfunction, providing a future direction for study of possible mechanisms. Evidence of skeletal muscle mitochondrial damage has not been previously reported in animal models of postnatal hyperoxia exposure. Our results raise two important questions. First, what is the mechanism behind the muscle dysfunction and mitochondrial damage in a rat model of postnatal hyperoxia exposure; and second, is the mitochondrial dysfunction identified in our model present in patients with a history of preterm birth. Future research exploring whether the rodent model of postnatal hyperoxia reproduces the metabolic and contractile disease seen in humans born preterm may shed light on the mechanism behind increased muscle fatigue and mitochondrial dysfunction in patients born premature as they age into adulthood. Lastly, although we showed similar results in skeletal muscle to previous reports of insulin resistance, we did not perform a specific assay to answer that question. Future work including a glucose tolerance test along with fasting insulin and glucose measures will provide insight into whether the rat model of postnatal hyperoxia recapitulates the insulin resistance found in human adults born preterm.

In summary, we found decreased muscle function which correlated with decreased mitochondrial function in skeletal muscle accompanied by compensatory increases in glycolytic capacity in the equivalent of middle aged male rats exposed to postnatal hyperoxia. Systemic mitochondrial metabolism, specifically in the skeletal muscle, should be further studied in animal models and patients with a history of preterm birth, as the implications in patients for developing long-term morbidities such as decreased exercise capacity, insulin resistance, and metabolic syndrome may be significant. Moreover, awareness of the potential differences between males and females born preterm can aid in the care of these patients, and providers should consider the possible increased risk for impaired exercise tolerance and metabolic disease in males.

## Author contributions

LT, GD, ME, and KG: Contributed to study conception and design; LT, GD, RB, HY: Contributed to data collection; LT, GD, GB, RB, HY, KH, ME, and KG: Contributed to data analysis and interpretation; LT: Drafted the initial manuscript; LT, GD, GB, RB, HY, KH, ME, and KG: Contributed to manuscript editing.

### Conflict of interest statement

The authors declare that the research was conducted in the absence of any commercial or financial relationships that could be construed as a potential conflict of interest.

## Supplementary material

Supplemental Figure 1 Images of all described western blots. Protein bands are above their respective loading control.
