# Supplementary figures and images for: Sex-Specific Skeletal Muscle Fatigability and Decreased Mitochondrial Oxidative Capacity in Adult Rats Exposed to Postnatal Hyperoxia
Source: Front Physiol. 2018 Mar 29;9:326. doi: 10.3389/fphys.2018.00326 (PMC5884929; doi:10.3389/fphys.2018.00326)

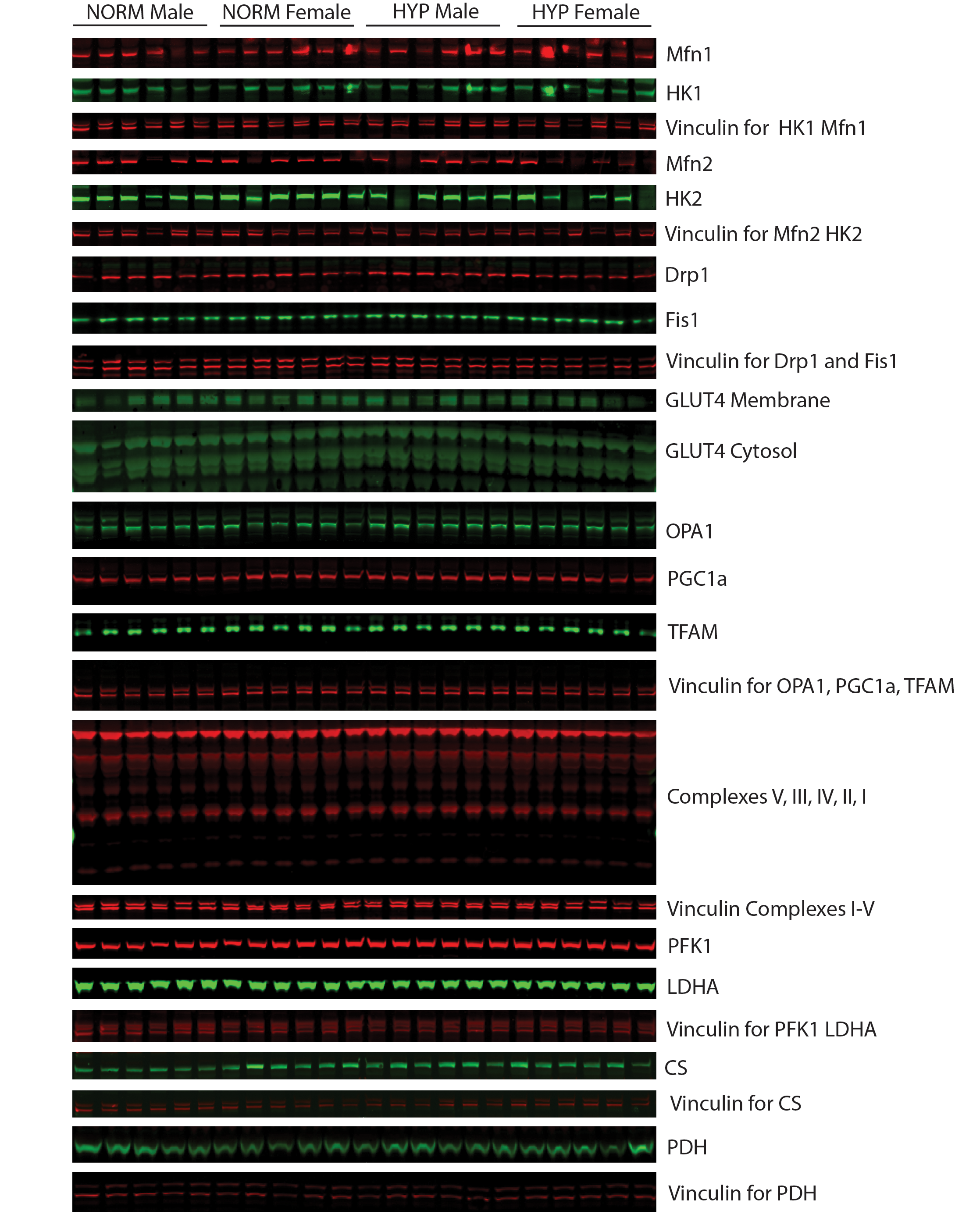

Supplement: Supplemental Figure 1 — Images of all described western blots. Protein bands are above their respective loading control. [file Image1.TIF]
